# Fermented soybean meal modified the rumen microbiota and increased the serum prolactin level in lactating Holstein cows

**DOI:** 10.3389/fvets.2024.1498639

**Published:** 2024-11-13

**Authors:** Jiyou Zhang, Feng Guan, Shana Huang, Yumin Ma, Shibao Wen, Wei Jin, Shengyong Mao

**Affiliations:** ^1^Laboratory of Gastrointestinal Microbiology, College of Animal Science and Technology, Nanjing Agricultural University, Nanjing, China; ^2^Inner Mongolia Agriculture and Animal Husbandry Technology Extension Center, Hohhot, China; ^3^Jiangsu Jiahui Biotechnology Co., Ltd., Haian, China; ^4^Shanghai Menon Animal Nutrition Technology Co., Ltd., Shanghai, China

**Keywords:** fermented soybean meal, cows, milk performance, serum biochemical indices, rumen fermentation, bacterial community

## Abstract

This study aimed to investigate the effects of fermented soybean meal (FSM) on milk production, blood parameters, and rumen fermentation and microbial community in dairy cows. In this study, 48 healthy Holstein cows (parity, 3.0 ± 0.6; days in milk, 86.0 ± 6.7) were used. Cows were randomly assigned into four groups (CON, T-200, T-400, and T-600) with 12 cows per group. Cows in CON were not supplemented with FSM. Cows in T-200, T-400, and T-600 were supplemented with 200, 400, and 600 g/head/day FSM, respectively. This study lasted 5 weeks (1-week adaptation and 4-week treatment). The results showed that FSM did not affect milk yield and milk components (*p* > 0.05). In the serum, FSM greatly increased prolactin (*p* < 0.01), and a dosage effect was observed. Aspartate aminotransferase and total protein were the highest in the T-400 (*p* < 0.05), and triglycerides was the lowest in T-200 (*p* < 0.05), and there was no difference for the 3 measurements between the other 3 groups (*p* > 0.05). In the rumen, FSM did not affect pH, microbial crude protein, acetate, propionate, butyrate, valerate, total volatile fatty acids and the ratio of acetate:propionate (*p* > 0.05), only changed NH_3_-N, isobutyrate and isovalerate (*p* < 0.05). The results of the rumen bacterial 16S rRNA genes sequencing showed that FSM decreased the richness (*p* < 0.05) and evenness (*p* < 0.05) of the bacterial communities. PCoA analysis showed that FSH altered the rumen bacterial community (ANOSIM, *R* = 0.108, *p* = 0.002). In the relative abundance of phyla, FSM increased Firmicutes (*p* = 0.015) and Actinobacteriota (*p* < 0.01) and Patescibacteria (*p* = 0.012), decreased Bacteroidota (*p* = 0.024). In the relative abundance of genera, FSM increased Christensenellaceae R-7 group (*p* = 0.011), *Lactococcus* (*p* < 0.01), *Candidatus Saccharimonas* (*p* < 0.01), *Olsenella* (*p* < 0.01), decreased Muribaculaceae_norank (*p* < 0.01). Conclusively, supplemented FSM altered the rumen fermentation parameters and bacterial community, and increased serum prolactin level in lactating Holstein cows. These findings may provide an approach to keep the peak of lactation in dairy cows.

## 1 Introduction

Fermented soybean meal (FSM) is a high-quality plant protein source for animals, containing probiotics, digestive enzymes, bioactive peptides, antioxidants and providing immunomodulatory effects (1). Many studies reported that feeding FSM to animals (pigs, chicken and calves) showed positive effects with improved nutrient digestibility and intestinal health and production performance (2–4). Due to the ban on the use of antimicrobial growth promoters in animal production, the use of FSM in ruminants has attracted a great interest.

In the study of Kim et al. (5), FSM had been used in a calf starter and showed positive effects on the health and growth of calves. As demonstrated by Feizi et al. (4), FSM improved the starter intake in calves, and altered the rumen fermentation and microbiota. In another study of Rezazadeh et al. (6), feeding FSM helped calves adapt to weaning stress during cold weather. One study in lactating cows reported that feeding FSM increased milk protein yield, milk fat yield and fat corrected milk, and decreased milk somatic cell count (7). Studies also showed that feeding FSM alter the rumen fermentation parameters and rumen microbiota in lactating Holstein cows (7, 8). However, the results were not consistent. As reported by Wang et al. (8), FSM reduced total volatile fatty acid concentration, acetate to propionate ratio and increased propionate percentage. According to Amin et al. (7), FSM increased rumen pH, acetate percentage and acetate to propionate ratio. Most studies regarding the use of FSM in ruminants have been focused on calves, few studies investigated the lactating cows, especially for cows in early stage of lactation (1).

We hypothesized that feeding FSM could cause changes in the rumen fermentation and microbiota and blood parameters which could lead to improve in the milk performance of dairy cows. In this study, we aimed to explore the effects of feeding FSM on the milk performance, blood parameters, and rumen fermentation and bacterial community in dairy cows in the early stage of lactation. The results would offer a reference for the application of FSM in the dairy cow industry.

## 2 Materials and methods

### 2.1 Animals, diets, and management

This experiment was conducted from November 2020 to December 2020 at Shanghai Jinshan Yinan Dairy Farm (Shanghai, China). In this study, 48 healthy Holstein cows in similar parity (3.0 ± 0.6) and lactation stages (86.0 ± 6.7 day in milk) and milk yield (41.0 ± 2.8 kg) were used. Cows were randomly assigned into 4 groups (CON, T-200, T-400, and T-600) with 12 cows per group. Cows in CON were not supplemented with FSM. Cows in T-200, T-400, and T-600 were supplemented with 200, 400, and 600 g/head/day FSM, respectively. This study lasted 5 weeks (1-week adaptation and 4-week treatment). FSM (yellow granular substance, fermented by *Saccharomyces cerevisiae* and *Bacillus subtilis*) used in this study was purchased from Shanghai Yuanyao Agriculture Co., Ltd. The basic diet used in this study was formulated based on NRC (2001) guidelines for lactating cows. The nutritional composition of FSM was shown in Supplementary Table S1. The ingredients and chemical composition of the basic diet were shown in Supplementary Table S2. All cows were housed in a tie stall barn, milked three times daily using a fully automated pipeline milking machine (02,30, 10,30, 16:30), and fed with total mixed ration three times daily (03,30, 10,30, 16:30), ensuring that cows had at least 20 h of free access to feed per day and free access to fresh water.

### 2.2 Sampling

Milk yield was determined by a Tunisian flow-meter (JHF-G17, Sichuan Jinhaifeng Animal Husbandry Technology Co., Ltd., Sichuan, China). Milk samples were collected at the last 2 days in each week, and preserved with potassium dichromate, at 4°C. Milk samples collected from the morning, afternoon, and evening milking daily were mixed at a ratio of 4:3:3 before determining the milk composition using a near-infrared analyzer (MilkoScanTM 7 RM, Foss Electric, Denmark).

The blood samples were collected via the tail vein of the cows before morning feeding on the last day of the trial. The collected blood samples were immersed in warm water (37°C) for 10 min immediately before centrifuging at 3,500 r/min for 15 min. The supernatant was collected and stored at −20°C for the determination of serum biochemical indices.

The rumen content samples were collected at 4 h after morning feeding using an oral ruminal tube (Wuhan Kelibao Co., Ltd., Wuhan, China) on the last day of the trial. In order to avoid saliva contamination, the first 200 mL rumen fluid was discarded. A portion of the rumen content was stored in liquid nitrogen for the measuring the microbial community. Another portion was filtered through four layers of sterilized cheesecloth, and stored at −20°C for the determination of microbial crude protein, NH_3_-N, and volatile fatty acids.

### 2.3 Chemical analysis

The serum biochemical indices were measured using a fully automated biochemical analyzer (Vital Scientific NV, The Netherlands) following the standard procedure. Prolactin (PRL) is a milk-production hormone, was measured using an enzyme-linked immunosorbent assay kit (ELISA kit, Shanghai, China).

The pH value of rumen fluid was measured using a portable pH meter (HI 9024C; HANNA Instruments, Woonsock, RI). The concentration of NH_3_-N in rumen fluid was determined using a phenol sodium hypochlorite colorimetric method according to Weatherburn (9). The microbial crude protein (MCP) content in rumen fluid was determined using a Coomassie Brilliant Blue colorimetric method according to Makkar et al. (10). The volatile fatty acids (VFA) concentration in rumen fluid was determined by a gas chromatography (GC-2014B, Shimadzu, Japan) equipped with a capillary column (column temperature: 110°C, film thickness: 30 m × 0.32 mm × 0.25 μm) (11).

### 2.4 Rumen microbial genomic DNA extraction

Rumen microbial genomic DNA was extracted using a phenol-chloroform extraction and cell lysis methods (12). The concentration of DNA was measured by a Nanodrop spectrophotometer (Nyxor Biotech; Paris, France) and stored at −80°C for further sequencing.

### 2.5 MiSeq sequencing

A pair of PCR primers was used to amplify the V3-V4 region of the rumen bacterial 16S rRNA genes (13). The primers were 341F (5-CCTAYGGGGRBGCASCAG-3) and 806R (5-GGACTACNNGGGTATCTAAT-3). The amplicons were sequenced on an Illumina MiSeq PE 300 platform (Illumina Inc., San Diego, California, United States) in a commercial laboratory (Shanghai Biozeron Technology Co., Ltd., Shanghai, China). The raw data were stored in the Sequence Read Archive (SRA) database, the accession number is PRJNA1162692.

### 2.6 Data analysis

Trimmomatic (v.0.33) software was used to trim adapters and low-quality sequences. FLASH (1.2.7) software was utilized to concatenate paired segments into a sequence (14). A software (QIIME2 v1.9.0) was used to process the raw Illumina fastq files (15). Bases with an average quality value below 20 were filtered. UPARSE software was used to classify sequences with a similarity level ≥ 97% into OTUs (16). The SILVA database was used to perform the taxonomic assignment of the representative OTU sequences (17). Principal coordinate analysis (PCoA) was conducted based on the Bray–Curtis metrics (18). The differences among groups was evaluated by ANOSIM using the vegan package in R.

### 2.7 Statistical analysis

A SPSS 20.0 software (SPSS Inc., Chicago, IL, United States) was used to analyze the data in this study.

The data (milk yield, and components) were analyzed with repeated measurements using a MIXED procedure, and adjusted with the data of adaption period as a covariate factor. The model included the fixed effects of treatment (CON, T-200, T-400, and T-600), time (week 1 to 4), treatment × time, and covariate. Time (week) was used as a repeated measurement with cows as the subject.

Data (rumen fermentation parameters, serum biochemical indices) were analyzed using the one-way ANOVA test. Significant difference between treatments was evaluated using Duncan’s test. Data on bacterial communities were analyzed using the nonparametric test (Kruskal-Wallis). Significance was declared at *p* < 0.05. All results were expressed as mean ± standard error.

## 3 Results

### 3.1 Milk yield and milk composition

As shown in Table 1, there were no effects of treatment (*p* > 0.05), time (*p* > 0.05), and treatment by time (*p* > 0.05) for milk yield, milk fat percentage, total milk solids, and somatic cell count. There were effects of time (*p* < 0.05), but not treatment (*p* > 0.05) and treatment by time (*p* > 0.05) for milk protein percentage, milk lactose percentage or milk urea nitrogen concentration.

**Table 1:** Effects of feeding FSM on milk yield and milk composition in lactating cows.

	Treatment					*p*-value		
Items	CON	T-200	T-400	T-600	SEM	Treatment	Time	Treatment × Time
Milk yield (kg)	40.85	40.90	42.00	41.45	0.42	0.498	0.638	0.217
Milk fat (%)	3.83	3.91	3.56	3.69	0.06	0.569	0.170	0.310
Milk protein (%)	3.13	3.02	3.19	3.23	0.03	0.146	0.001	0.950
Milk lactose (%)	5.28	5.23	5.29	5.33	0.02	0.546	<0.001	0.085
Total milk solids (%)	12.55	12.51	12.36	12.55	0.08	0.471	0.253	0.470
Somatic cell count (10^3^/mL)	59.08	105.50	158.46	70.83	36.18	0.528	0.826	0.384
Milk urea nitrogen (g/L)	15.65	16.04	16.06	17.85	0.26	0.572	<0.001	0.421

### 3.2 Serum biochemical indices

As shown in Table 2, there were treatment effects for PRL (prolactin), AST (Aspartate aminotransferase), TP (Total Protein), TRIG (Triglycerides) in the serum (*p* < 0.05). PRL showed a dosage effect, and increased with the dosage increase of FSM (394.67, 493.81, 536.16, and 608.13 mIU/L, *p* < 0.01). AST was higher in T-400 (*p* < 0.05), and did not differ between the other 3 groups (*p* > 0.05). TP was higher in T-400 than that in CON and T-200 (*p* < 0.05), and T-400 did not differ with T-600 (*p* > 0.05). TRIG was lower in T-200 than that in CON and T-600 (*p* < 0.05), and T-200 did not differ with T-400 (*p* > 0.05). There were no treatment effects for T-SOD (superoxide dismutase), ALT (Alanine aminotransferase), ALB (Albumin), ALP (Alkaline Phosphatase), GLOB (Globulin), A/G (Albumin/ Globulin), ALP (Alkaline Phosphatase), CK (Creatine Kinase), LDH (Lactic Acid Dehydrogenase), HDL-C (High-density lipoprotein), LDL-C (Low-density lipoprotein), CREAT (Creatinine), TCHO (Total cholesterol), GLU (Glucose), and UA (uric acid) in the serum (*p* > 0.05).

**Table 2:** Effects of feeding FSM on serum biochemical indices in lactating cows.

	Treatment					
Items	CON	T-200	T-400	T-600	SEM	*P*-value
T-SOD(U/mL)	100.17	105.75	107.33	109.92	6.57	0.758
AST (U/L)	78.08^b^	79.67^b^	94.83^a^	74.58^b^	4.83	0.025
ALT (U/L)	32.08	33.08	35.75	33.42	1.59	0.425
TP (g/L)	73.19^b^	73.08^b^	76.66^a^	75.74^ab^	0.99	0.027
ALB(g/L)	37.33	37.88	37.98	37.87	0.44	0.710
GLOB(g/L)	35.86	35.21	38.68	37.87	1.02	0.064
A/G	1.04	1.08	0.98	1.02	0.04	0.184
ALP(U/L)	60.83	69.42	70.58	76.75	4.94	0.168
CK(U/L)	204.50	155.67	318.25	164.67	70.74	0.936
LDH (U/L)	923.75	918.00	952.17	915.58	32.92	0.852
UREA (mmol/L)	5.10	4.73	4.87	5.03	0.18	0.482
CREA (μmol/L)	58.98	61.58	57.07	60.19	1.65	0.273
GLU (mmol/L)	2.45	2.35	2.42	2.71	0.11	0.114
UA (μmol/L)	58.50	58.50	60.00	54.00	2.76	0.339
TCHO (mmol/L)	6.52	6.35	6.90	6.30	0.30	0.481
TRIG (mmol/L)	0.24^a^	0.15^b^	0.20^ab^	0.23^a^	0.02	0.012
HDL-C (mmol/L)	1.78	1.89	1.64	1.44	0.13	0.085
LDL-C (mmol/L)	2.48	2.23	2.49	2.30	0.15	0.524
PRL(mIU/L)	394.67^c^	493.81^b^	536.16^ab^	608.13^a^	26.68	<0.01

AST, Aspartate aminotransferase; ALT, Alanine aminotransferase; TP, Total Protein; ALB, Albumin; ALP, Alkaline Phosphatase; GLOB, Globulin; A/G, Albumin/ Globulin; ALP, Alkaline Phosphatase; CK, Creatine Kinase; LDH, Lactic Acid Dehydrogenase; HDL-C, High-density lipoprotein; LDL-C, Low-density lipoprotein; CREAT, Creatinine; TCHO, Total cholesterol; TRIG, Triglycerides; GLU, Glucose; PRL, prolactin; T-SOD, superoxide dismutase; UA, uric acid. a, b, cWithin a row, mean values with different superscript letters indicate a significant difference (*P* < 0.05).

### 3.3 Rumen fermentation parameters

As shown in Table 3, there were no treatment effects for rumen pH, microbial crude protein, acetate, propionate, butyrate, valerate, total volatile fatty acids and the ratio of acetate to propionate (*p* > 0.05). There were treatment effects for the concentration of NH_3_-N, isobutyrate, and isovalerate (*p* < 0.05). The concentration of NH_3_-N was higher in T-400 than the other 3 groups (*p* < 0.05). Isobutyrate was lower in T-200 and T-400 than that in CON (*p* < 0.05), but not differ with that in T-600 (*p* > 0.05). Isovalerate was lower in T-400 than that in CON and T-600 (*p* < 0.05), but not differ with that in T-200 (*p* > 0.05).

**Table 3:** Effects of feeding FSM on rumen fermentation parameters in lactating cows.

	Treatment					
Items	CON	T-200	T-400	T-600	SEM	*P*-value
Ruminal pH	6.28	6.33	6.21	6.33	0.09	0.727
NH_3_-N (mg/dL)	12.05^b^	13.11^b^	16.86^a^	13.57^b^	1.01	0.011
Microbial crude protein (mg/dL)	39.69	39.74	34.39	33.89	2.99	0.335
Acetate (mmol/L)	77.17	73.81	76.25	78.84	2.61	0.590
Propionate (mmol/L)	29.84	26.06	27.55	26.43	1.66	0.378
Isobutyrate (mmol/L)	1.08^a^	0.87^b^	0.85^b^	0.97^ab^	0.06	0.026
Butyrate (mmol/L)	14.20	13.93	14.76	15.42	0.60	0.310
Isovalerate (mmol/L)	1.61^a^	1.39^ab^	1.30^b^	1.62^a^	0.09	0.036
Valerate (mmol/L)	1.81	1.66	1.71	1.83	0.13	0.769
Total volatile fatty acids (mmol/L)	125.70	117.72	122.42	125.11	4.30	0.546
Acetate: Propionate	2.64	2.92	2.86	3.05	0.13	0.188

a, b, cWithin a row, mean values with different superscript letters indicate a significant difference (*P* < 0.05).

### 3.4 Rumen bacterial community

**Figure 1:**
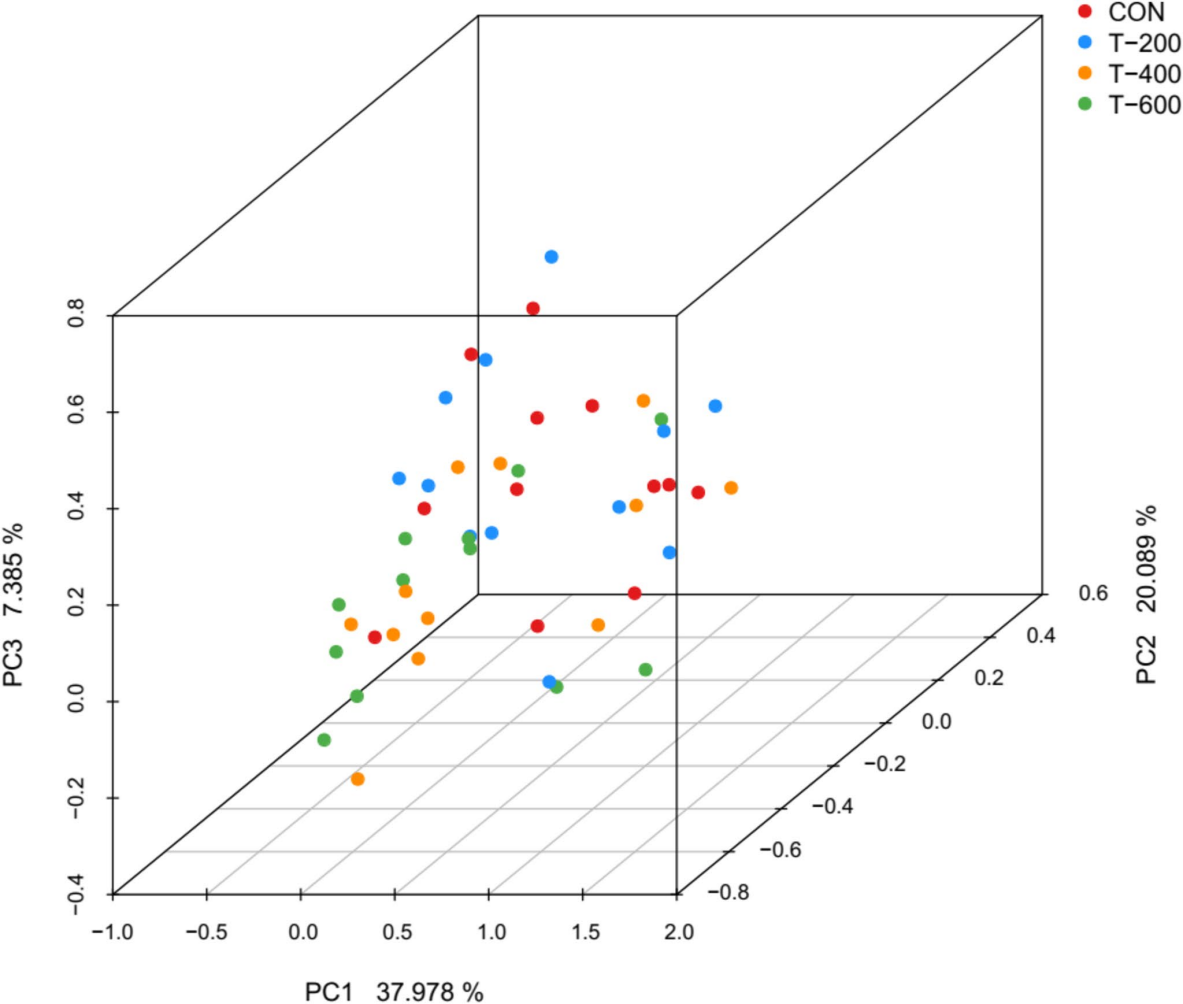
Principal coordinate analysis (PCoA) plot of ruminal bacterial communities based on the Bray Curtis metrics.

There were total of 2,128,625 high-quality sequences were obtained from 48 samples, with an average of 44,346 sequences for each sample. The rarefaction curve tended to flatten out, indicating that the sequencing depth were sufficient for analyzing the rumen bacterial communities (Supplementary Figure S1). PCoA based on the Bray Curtis metric algorithm showed that FSM altered the rumen bacterial community structure (ANOSIM: *R* = 0.108, *p* = 0.002) (Figure 1). Significant differences were observed between CON and T-400 (*R* = 0.074, *p* = 0.032); CON and T-600 (*R* = 0.109, *p* = 0.004); T-200 and T-400 (*R* = 0.076, *p* = 0.018); T-200 and T-600 (*R* = 0.103, *p* = 0.003). There were no differences between CON and T-200 (*R* = 0.042, *p* = 0.441); T-400 and T-600 (*R* = 0.039, *p* = 0.539).

As shown in Table 4, there were treatment effects for the number of OTUs (*p* < 0.01), Chao 1 index (*p* = 0.043), and Shannon index (*p* = 0.028). The number of OTUs and Chao 1 and Shannon were lower in T-400 and T-600 than that in CON (*p* < 0.05). There were no treatment effects for Simpson (*p* = 0.051).

**Table 4:** Effects of feeding FSM on alpha diversity of rumen bacterial community in lactating cows.

	Treatment					
Items	CON	T-200	T-400	T-600	SEM	*P*-value
OTU	3389^a^	3179^ab^	2882^b^	2931^b^	96	<0.01
Chao 1	4779^a^	4400^ab^	4145^b^	4186^b^	145	0.043
Shannon	6.682^a^	6.64^ab^	6.47^b^	6.48^b^	0.06	0.028
Simpson	0.0046	0.0044	0.0059	0.0066	0.001	0.051

a, b, cWithin a row, mean values with different superscript letters indicate a significant difference (*P* < 0.05).

As shown the relative abundance of phyla in Table 5, there were treatment effects for Firmicutes (*p* = 0.015), Bacteroidota (*p* = 0.024), Actinobacterota (*p* < 0.01), and Patescibacteria (*p* = 0.012). Firmicutes was higher in T-400 and T-600 than that in CON and T-200 (*p* < 0.05). Bacteroidota was lower in T-400 and T-600 than that in CON and T-200 (*p* < 0.05).

**Table 5:** Effects of feeding FSM on the relative abundance of phyla of rumen bacterial community in lactating cows.

	Relative abundance, %					
Phylum	CON	T-200	T-400	T-600	SEM	*P*-value
Firmicutes	42.19^b^	40.93^b^	49.13^a^	51.36^a^	1.27	0.015
Bacteroidota	48.34^a^	48.88^a^	40.54^b^	39.01^b^	1.38	0.024
Proteobacteria	1.01	1.62	1.72	2.05	0.22	0.290
Euryarchaeota	3.24	3.29	2.27	1.87	0.23	0.081
Actinobacteriota	0.97^c^	1.25^bc^	2.26^a^	1.74^ab^	0.12	<0.01
Spirochaetota	1.58	1.25	1.12	0.90	0.09	0.103
Patescibacteria	1.37^b^	1.51^b^	1.59^b^	2.00^a^	0.06	0.012

a, b, cWithin a row, mean values with different superscript letters indicate a significant difference (*P* < 0.05).

Actinobacterota was higher in T-400 and T-600 than that in CON (*p* < 0.05). Patescibacteria was higher in T-600 than that in the other 3 groups (*p* < 0.05).

As shown the relative abundance of genera in Table 6, there were treatment effects for *Muribaculaceae_norank* (*p* < 0.01), *Christensenellaceae R-7 group* (*p* = 0.011), *Lactococcus* (*p* < 0.01), *Candidatus Saccharimonas* (*p* < 0.01), and *Olsenella* (*p* < 0.01). *Muribaculaceae_norank* was lower in T-400 and T-600 than that in CON (*p* < 0.05). *Christensenellaceae R-7 group* was higher in T-600 than that in CON and T-200 (*p* < 0.05). *Lactococcus* and *Candidatus Saccharimonas* were higher in T-600 than that in the other 3 groups (*p* < 0.05). *Olsenella* was higher in T-400 and T-600 than that in CON (*p* < 0.05).

**Table 6:** Effects of feeding FSM on the relative abundance of genera of rumen bacterial community in lactating cows*.

		Treatment					
Phylum	Genus	CON	T-200	T-400	T-600	SEM	*P*-value
*Bacteroidota*	*Prevotella*	24.86	25.97	21.18	19.27	1.44	0.233
	*Rikenellaceae RC9 gut group*	5.73	6.15	5.36	6.51	0.30	0.586
	*Muribaculaceae_norank*	5.69 ^a^	4.72 ^ab^	3.50 ^b^	3.39 ^b^	0.31	<0.01
	*Prevotella_7*	1.85	1.06	1.12	0.35	0.29	0.131
	*F082_norank*	3.42	4.2	3.84	4.81	0.20	0.076
	*Bacteroidales RF16 group_norank*	1.46	1.39	1.12	1.30	0.10	0.735
	*Prevotellaceae YAB2003 group*	0.37	0.65	0.33	0.16	0.066	0.301
	*Prevotellaceae UCG-003*	1.13	1.29	0.95	0.87	0.062	0.154
*Firmicutes*	*Succiniclasticum*	6.61	5.83	7.19	8.23	0.54	0.626
	*NK4A214 group*	6.67	5.91	7.93	7.93	0.38	0.143
	*Ruminococcus*	3.54	4.26	4.35	5.08	0.31	0.405
	*Christensenellaceae R-7 group*	3.99 ^bc^	3.75 ^c^	5.42 ^ab^	6.35 ^a^	0.31	0.011
	*Clostridia UCG-014_norank*	2.52	2.52	2.74	2.18	0.20	0.254
	*Lachnospiraceae NK3A20 group*	2.62	2.13	3.49	2.96	0.21	0.066
	*[Ruminococcus] gauvreauii group*	0.54	0.53	0.88	0.60	0.10	0.613
	*UCG-005*	0.91	0.61	0.39	0.51	0.089	0.084
	*Butyrivibrio*	0.60	1.06	0.86	1.20	0.094	0.355
	*Acetitomaculum*	1.30	1.32	1.80	2.05	0.097	0.210
	*Lactococcus*	0.005 ^b^	0.017 ^b^	0.18 ^b^	0.54 ^a^	0.065	<0.01
*Proteobacteria*	*Acinetobacter*	0.012	0.025	0.39	0.88	0.180	0.056
*Spirochaetota*	*Treponema*	1.56	1.24	1.10	0.89	0.093	0.107
*Patescibacteria*	*Candidatus Saccharimonas*	1.19 ^c^	1.34 ^bc^	1.53 ^b^	1.96 ^a^	0.067	<0.01
*Actinobacteriota*	*Bifidobacterium*	0.23	0.40	0.76	0.53	0.087	0.108
	*Olsenella*	0.43 ^c^	0.52 ^bc^	0.97 ^a^	0.65 ^ab^	0.062	<0.01

*Genera whose relative abundance >0.5% in at least one group were shown. a, b, cWithin a row, mean values with different superscript letters indicate a significant difference (*P* < 0.05).

## 4 Discussion

FSM is a high-quality protein, containing probiotics, digestive enzymes, bioactive peptides, antioxidants, and low-antinutritional-factors. Feeding FSM would provide positive effects on dairy cows, especially for the cows in the early stage of lactation, during which cows suffer multiple stress. In this study, feeding FSM did not affect the milk yield and milk composition, just observed a numerical increase in milk yield, milk protein percentage and milk urea nitrogen. It is not consistent with a previous study. Amin et al. (7) reported that feeding FSM increased the milk protein yield, milk fat yield and fat corrected milk, and decreased milk somatic cell count in cows in early lactation stage (54 days in milk). The different results observed may be attributed to the varying dosages of FSM supplementation, the inoculum used, the composition of the basic diets, and the lactation stages of cows involved in the different studies (1). Studies regarding FSM on lactating cows are very few, thus more works are needed to elucidate the action mode of FSM in lactating cows.

Serum biochemical indices are indirect indicators of the health and metabolic status of livestock. Feeding FSM caused a little change in a few blood measurements, but caused a great increase in the serum prolactin concentration. These changes were not observed or not measured in the previous studies of Amin et al. (7) and Wang et al. (8). Prolactin is an important lactation hormone that plays a crucial role in promoting mammary gland development, milk synthesis, milk yield, and maintaining lactation (19). During milk synthesis, prolactin facilitates the absorption of glucose and amino acids, as well as the synthesis of milk lactose, fat, casein, and lactoglobulin (20). The mechanism underlying the increase of serum prolactin by feeding FSM is still unclear. In this study, FSM treatment only last 4 weeks, the effect of maintenance of lactation did not exhibited. Further studies would last 8 weeks or longer to explore the effect of FSM on the lactation maintenance in lactating cows. Nevertheless, the new finding might provide a new strategy for the utilization of FSM on lactating cows, especially in the early lactation stage.

Feeding FSM did not affect the concentration of acetate, propionate, butyrate, total volatile fatty acids in the rumen, only caused a little change in concentration of NH_3_-N, isobutyrate and isovalerate. Two previous studies reported that FSM changed the rumen fermentation parameters in lactating Holstein cows (7, 8). Wang et al. (8) reported that FSM reduced total volatile fatty acid concentration, acetate to propionate ratio and increased propionate percentage. Amin et al. (7) reported that FSM increased rumen pH, acetate percentage and acetate to propionate ratio. The results from these studies were not consistent. The underlying reasons remain to elucidate.

Feeding FSM caused changes in the rumen bacterial community. The previous studies also reported that feeding FSM modified the rumen bacterial communities in lactating Holstein cows (7, 8). However, the changes in these studies were not consistent. In the current study, feeding FSM decreased the number of OTUs, Chao 1 and Shannon indexes, and increased the relative abundance of Firmicutes and decreased the relative abundance of Bacteroidota in the phylum levels, which were not observed in the two previous studies. Amin et al. (7) reported that FSM enriched the genus of *Muribaculaceae_norank*, which were reduced in the current study. Both the current study and the study of Amin et al. (7) observed the enrichment of the genus of *Christensenellaceae R-7 group* by feeding FSM. The enrichment of the genera of *Lactococcus*, *Candidatus Saccharimonas* and *Olsenella* was not observed in the two previous studies. Fernando et al. (21) reported that *Firmicutes* were more adapted to fiber fermentation, while *Bacteroidota* was more effective in degrading starch. Wang et al. (22) reported that *Actinobacteriota* had the ability to degrade polysaccharides. It suggested that FSM enhanced the fiber fermentation ability in the rumen. Lagkouvardos et al. (23) reported that the genomes of *Muribaculaceae* contained a substantial and versatile set of carbohydrate-active enzymes, suggesting that the members in this family had the ability to degrade complex carbohydrates, the authors also stated that the fitness of *Muribaculaceae* species in degrading dietary carbohydrates most likely explains the decreased occurrence in the feeding trials using high-calories or carbohydrate-enriched diets. *Lactococcus* are homofermentative and are used for the production of L(+) lactic acid from glucose. In dairy industry, *Lactococcus* species are used majorly in the production of lactic acid from lactose, hydrolysis of casein, fat lipolysis by weak esterase activities, and citric acid fermentation (24). The enrichment of *Lactococcus* might be due to the enhancement of the fiber degradation or due to the nutrients provided by FSM. The enrichment of *Candidatus Saccharimonas* in the rumen was observed by feeding a *Saccharomyces cerevisiae* fermentation product in lactating Holstein cows (25). Tong et al. (26) reported that the abundance of the *Candidatus Saccharimonas* was positively correlated with the concentration of propionate in the rumen of lactating cows. Ranilla et al. (27) observed that an antioxidant (carvacrol) enriched *Candidatus Saccharimonas* in an *in vitro* trial. It suggested that the enrichment of *Candidatus Saccharimonas* might be associated with the antioxidant provided by FSM. The members of *Olsenella* could utilize starch and glycogen, producing lactate, acetate, and formate (28). Kim et al. (29) reported that the relative abundance of *Olsenella* was higher in the rumen of Holstein cows fed a high-grain diet. McLoughlin et al. (30) reported that the relative abundance of *Olsenella* in the rumen was positively associated with feed efficiency in sheep. Elolimy et al. (31) observed a higher relative abundance of *Olsenella* in the hindgut of Holstein heifer calves with high feed efficiency. However, Ellison et al. (32) found a higher abundance of *Olsenella* in the rumen of low feed efficient lambs fed a concentrate diet. It suggested that FSM increased the feed efficiency in the current study. Unfortunately, the feed efficiency was not determined in this study. It should be measured in the further study.

FSM is a high-quality plant protein source containing more than 50% crude protein. The study supplemented FSM directly into the diets without modifying the dietary protein levels across the various treatment groups. As a result, the dietary crude protein levels in the treatment groups increased by approximately 0.7 to 1.4% compared to the control group. A slight rise was observed in the numeric value of the milk protein percentage and milk urea nitrogen, but this increase was statistically insignificant. The further studies would adjust the dietary protein levels to be the same across all treatments.

## 5 Conclusion

Feeding FSM to lactating cows did not affect the milk performance, but increased the serum prolactin levels which would help cows maintain the lactation. Moreover, feeding FSM only caused a minor change in rumen fermentation parameters, but greatly alter the rumen microbiota, with the increase of Firmicutes, and decrease of Bacteroidota in the relative abundance. Though more work should be done to demonstrate the effects of FSM, these findings may provide an approach to keep the peak of lactation in dairy cows.

## Data Availability

The datasets presented in this study can be found in online repositories. The names of the repository/repositories and accession number(s) can be found in the article/Supplementary material.
