# Supplementary material for: Fermented soybean meal modified the rumen microbiota and increased the serum prolactin level in lactating Holstein cows
Source: Front Vet Sci. 2024 Nov 13;11:1498639. doi: 10.3389/fvets.2024.1498639 (PMC11599173; doi:10.3389/fvets.2024.1498639)
Supplement: Supplementary file 1 [file Table_1.docx]

| Table S1 Nutritional content of fermented soybean meal（DM basis） | | |
| --- | --- | --- |
| Items |  | Content |
| Crude protein (%) |  | 58.15 |
| Soluble protein: Total protein Ratio (SP: CP) |  | 8.00 |
| Rumen digestible protein: Total protein (DP: CP) |  | 54.00 |
| Acid detergent fiber (%) |  | 8.88 |
| Neutral detergent fiber (%) |  | 14.19 |
| Non-fibrous carbohydrates (%) |  | 13.41 |
| Fat % |  | 5.08 |
| Ash % |  | 9.17 |
| Total digestible nutrient (TDN) |  | 80.00 |
| Net Energy for lactation (NEL) Mcal/Kg |  | 1.84 |
| Net energy for maintenance (NEM) Mcal/Kg |  | 1.95 |
| Net energy for Gain (NEG) Mcal/Kg |  | 1.30 |
| Calcium (Ca) |  | 0.46 |
| Phosphorus (P) |  | 0.99 |
| Magnesium (Mg) |  | 0.35 |
| Potassium (K) |  | 2.36 |
| Sulphur (S) |  | 0.42 |
| Dry Matter (%) |  | 94.80 |

Table S2 The ingredients and chemical composition of the basic diet (DM basis) %

| Items |  | Content |
| --- | --- | --- |
| Mixed concentrate^1^ (%) |  | 24.57 |
| Extruded soybean (%) |  | 2.19 |
| Soybean hull (%) |  | 2.19 |
| Whole Cottonseed (%) |  | 4.39 |
| Alfalfa (%) |  | 3.95 |
| Oat grass (%) |  | 3.29 |
| Alfalfa silage (%) |  | 6.58 |
| Corn silage (%) |  | 48.25 |
| Molasses (%) |  | 2.19 |
| Yeast XPC (%) |  | 0.07 |
| Fat powder (%) |  | 0.66 |
| Lysine (Lys) (%) |  | 0.02 |
| Baking soda (NaHCO_3_) (%) |  | 0.64 |
| Salt (NaCl) (%) |  | 1.21 |
| Methionine (Met) (%) |  | 0.07 |
| Urea (%) | 0.18 | |
| Premix I^2^ (%) | 1.32 | |
| Premix II^3^ (%) | 0.05 | |
| Premix III ^4^ (%) | 0.04 | |
| Total (%) |  | 100.00 |
| **Nutrient content** |  |  |
| Net Energy for lactation (NEL) (MJ/Kg) |  | 7.48 |
| CP |  | 17.50 |
| NDF |  | 36.78 |
| ADF |  | 21.25 |
| Ash |  | 8.56 |
| Calcium (Ca) |  | 1.24 |
| Phosphorus (P) |  | 0.71 |

NDF (Neutral detergent fiber), ADF (Acid detergent fiber), CP (crude protein),

Note: All parameters are analyzed values except the NEL which is calculated

^1^ One kilogram of mixed concentrate contains 700 g of Corn， and 300 g of Soybean meal.

^2^ One kilogram of premix I contains the following: Vitamin A (180 KIU)， Vitamin D (45 KIU)， Vitamin E (1400 KIU), copper methionine (150 mg), zinc methionine (700 mg), hydroxy analogue methionine (10 g), selenium yeast (20 mg), Cu (360 mg), Mn (910 mg), Fe (170 mg), Zn (680 mg), Se (6 mg), I (20 mg), Co (4 mg).

^3^ One kilogram of premix II contained the following: Zn 57.2g, Mn 28.6g, Cu 5g, Co1.4g

^4^ One kilogram of premix III contained the following： Vitamin C (10 g), Mg (20 g), K (5 g), Ca (2 g), Fe (10 g), Zn (10 g), Mn (1g), Cellulose enzyme (150000 IU), xylanase (200000 IU)


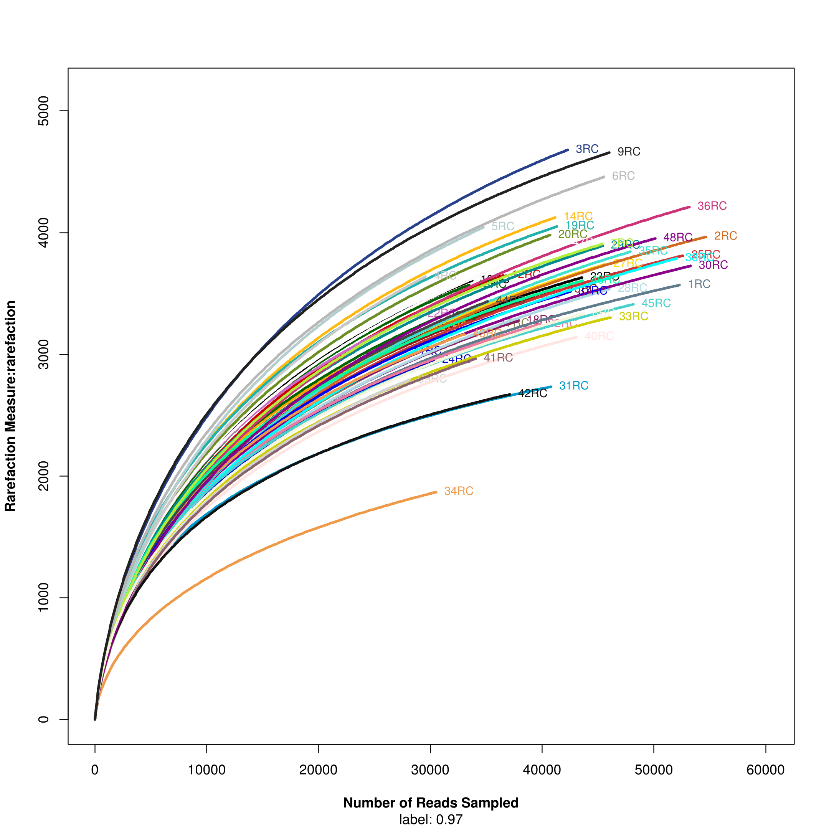


Figure S1 The rarefaction curves
